# A Systematic Review of Associations Between Interoception, Vagal Tone, and Emotional Regulation: Potential Applications for Mental Health, Wellbeing, Psychological Flexibility, and Chronic Conditions

**DOI:** 10.3389/fpsyg.2020.01792

**Published:** 2020-08-05

**Authors:** Thomas Pinna, Darren J. Edwards

**Affiliations:** Department of Public Health, Policy and Social Sciences, Swansea University, Swansea, United Kingdom

**Keywords:** emotional regulation, vagal tone, interoception, chronic conditions, health and well-being, psychological flexibility

## Abstract

**Background:** Interoception and heart rate variability have been found to predict outcomes of mental health and well-being. However, these have usually been investigated independently of one another.

**Objectives:** This systematic review aimed to explore a key gap in the current literature, that being, identifying whether HRV and interoception predict emotional regulation outcomes and strategies.

**Methods:** The process of article retrieval and selection followed the PRISMA guidelines. Databases PsychINFO, Web of Science, PubMed, CINAHL, and MEDLINE were scanned for papers published. Preliminary inclusion and exclusion criteria were specified following the PICO framework, whilst the CHARMS framework was used to help formulate the research question, and critically assess for bias.

**Results:** Two hundred and thirty-seven studies were identified after initial database searches. Of these, eight studies were included in the final selection. Six studies explored the associations between HRV and ER, whilst three investigated the associations between interoception and ER (one study included both). Results show that greater HRV and interoception are associated with better ER. Specifically, high parasympathetic activity largely predicted the use of adaptive ER strategies such as reappraisal, and better acceptance of emotions. High interoception, instead, was predictive of effective downregulation of negative emotions and handling of social uncertainty, there was no association with any specific ER strategy.

**Conclusions:** Awareness of one's own bodily feelings and vagal activation seem to be of central importance for the effective regulation of emotional responses. However, one limitation is the small sample of studies found, thus more studies in this area are needed in the future.

## Introduction

Emotions are a multifaceted construct involving overlapping aspects of the experiential, behavioral, and autonomic systems (Koole et al., 2011). It has been suggested that emotions drive behavioral modification when faced with constantly changing situations and direct individuals toward efficient, goal directed, behavioral responses (Damasio, 1999).

From an evolutionary perspective, emotions may provide a survival advantage as they are an important part of environmental adaptation, whether at a singular (benefiting the individual organism) (Cosmides and Tooby, 2000) or at a multi-level selection level (benefiting the group) (Wilson and Sober, 1994).

Emotions can be defined as self-regulatory, automatic, feeling processes that arise in response to internal, and external environmental stimuli (Thompson, 1994; Mauss et al., 2005), and these can be specific (e.g., angry about missing a bus) or global (e.g., feeling generally stressed and anxious) (Koole et al., 2011).

Both the autonomic nervous system (ANS) and endocrine system mediate allostatic processes necessary to allow for the efficient homeostatic levels necessary for survival (Kreibig, 2010; de Veld et al., 2012). For example, in an emotionally challenging situation, a person's hands may start sweating (to cool the body), their face may become red, their skin may tense, and their heart may beat faster (to ready them for a fight or flight response).

Despite these automated processes of the ANS, individuals have the ability to evaluate situations and adjust their frequency, latency, and intensity toward goal-oriented behavior (Gyurak et al., 2011). Lazarus (1991), one of the early theorists of emotions, defined two temporally distinct emotional responses. A primary response, which entails the raw, sensitivity-driven, response to an emotion-inducing event, and a secondary response, in which individuals are able to make judgements and regulate their emotional responses to cope with the primary response (Baumann et al., 2007).

Individuals have been shown to employ various cognitive-behavioral strategies to regulate emotions, some of which have been linked with positive and others with worse psychological outcomes (Aldao et al., 2010). The attempt to change the moment-to-moment emotional experiences, behavioral expressions, and physiological responses is called emotional regulation (ER) (Gross, 1998; Aldao, 2013). Effective ER is associated with better executive control, decision-making, understanding other's behaviors, and handling of social circumstances (van't Wout et al., 2010; Teper et al., 2013). From a perspective of physical health, more effective forms of ER are associated with reduced mortality, morbidity, cardiovascular issues, and better immune system function (Kiecolt-Glaser et al., 2002; Pressman and Cohen, 2005; Chida and Steptoe, 2009; Song and Chung, 2010). Emotional dysregulation, on the other hand, has been found to lead to inflexible behavioral and autonomic responses, the reinforcement of maladaptive behaviors and thoughts, increased inflammatory states, peripheral and central sensitization, and a negative effect on the overall bodily homeostatic balance (Bradley et al., 2011; Jazaieri et al., 2013).

Gross (1998) developed a process model of ER whereby he suggested that ER strategies can be broadly categorized as either antecedent-focused or response-focused. Antecedent-focused strategies were thought to be aimed at manipulating the inputs of emotion, for instance, by selecting situations with less emotional impact (situation selection), modifying the impact of a given environment (situation modification), shifting the attention away from the arousing event (attentional deployment) or revaluating the situation and thoughts held in relation to it (cognitive change). In contrast, response-focused strategies were thought to be aimed at manipulating the emotional output in order to intensify, diminish, or prolong it (Gross, 1998). Expressive suppression (such as thought suppression) is a response-focused strategy which is intended to be the active inhibition of uncomfortable emotions and thoughts. However, there is evidence that emotional suppression strategies are associated with greater cognitive distress and sympathetic arousal (Aldao et al., 2010). Other expressive suppression strategies such as using anti-anxiety drugs can often lead to the adverse effects of dizziness, headaches, and nausea (Gorman, 2003).

Antecedent-focused strategies are widely considered as the most adaptive ER strategies as they have been shown to provide better affective and physiological regulation with minimal cognitive and physical energetic expenditure (Ochsner and Gross, 2005; Buhle et al., 2014). One example of an antecedent strategy is emotional reappraisal which is the attempt to cognitively reframe an event to reduce its negative impact. This has shown to consistently activate executive function regions (pre-frontal, and temporal cortices) and modulate limbic activity of the amygdala in a beneficial way (Buhle et al., 2014).

Some evidence suggests that the ability to emotionally regulate successfully may be strongly dependent on the moment-to-moment awareness of bodily parameters relayed via interoceptive pathways which are measured through the use of an electrocardiogram (ECG) in the form of interoceptive awareness (IA) (Thayer and Lane, 2000; Craig, 2008). IA, more commonly referred to as interoception, refers to the internal representation of all bodily sensations in any given moment (Craig, 2002) and how the brain evaluates these sensations (Cameron, 2001). In addition to this, it is not limited to that of bodily sensations derived from the afferent component of the ANS, but also has a role in processing emotional, motivational, and behavioral outcomes (Craig, 2014; Owens et al., 2018). Damasio and Carvalho (2013) suggested that the interoceptive system is responsible for creating homeostatic maps of the body and orchestrating regulatory responses both at a conscious level, through emotions and feelings, and at the autonomic level.

Anatomically, the interoceptive system comprises of unmyelinated C and myelinated Aδ fibers converging signals into spinal laminae I and II, and reaching homeostatic centers, namely the hypothalamus, anterior insular and cingulate cortices (Pollatos et al., 2007). Interoceptive inputs stem from many physiological systems including the viscera, thermoregulatory, nociceptive, and endocrine systems. From there, inputs of varying motivational immediacy such as warmth-coldness, prickly-burning pain, taste, need to urinate, hunger, and sensual touch are relayed (Strigo and Craig, 2016). Importantly, these signals are integrated into primary emotional and motivational centers of the limbic system, the anterior insula and cingulate cortices (i.e., the homeostatic sensorimotor cortex), which have been shown to activate during all human emotions and motivational behaviors (Murphy et al., 2003; Craig, 2014). It is theorized that at these centers a meta-representation of the self is defined, allowing formulation of finely-tuned regulatory responses (Damasio and Carvalho, 2013).

In laboratory experiments, interoception is commonly measured as interoceptive accuracy (IAc), or sometimes called interoceptive sensitivity (IS) (these measures are the same). These measures are evaluated by asking participants to count their perceived heart beats which is a validated procedure called the heart beat perception task (Schandry, 1981; Pollatos et al., 2009). Numerous links have been established between altered IAc/IS and psychopathology, for instance in chronic pain, depression, anxiety, and eating disorders (Paulus and Stein, 2010; Klabunde et al., 2013; Di Lernia et al., 2016; Duschek et al., 2017). Generally, psychophysical pathology tends to be associated with reduced IAc/IS scores. It is suggested that the inefficient or impaired relay of interoceptive information from peripheral structures to the higher centers determines dysfunctional “body mapping” and, therefore, dysfunctional self-regulatory processes, with aberrant emotional, behavioral, and autonomic sequelae (Craig, 2008).

In addition to interoception, another psychophysiological factor which may influence the ability to emotionally regulate is the activity of the vagal nerve. The vagus nerve is the 10th cranial nerve (labeled CN X), it is the longest cranial nerve in the body, and the main anatomical component of the parasympathetic nervous system (Walker, 1990). Although often considered solely for its efferent functions, 80% of the fibers of the vagus relay sensory information from viscero-somatic structures (Porges, 2007a). Recently, the scope of the vagus has been expanded beyond visceral homeostatic functions to include social, cognitive, and affective components (Yuan and Silberstein, 2016). As the heart is under dominant tonic inhibitory control of the vagus nerve, electrocardiogram (ECG) measurements can be utilized in order to quantify vagal activity. This measure is an expression of interbeat variation, called Heart Rate Variability (HRV), that has been demonstrated to be a reliable indicator of several psychosomatic functions including emotional and behavioral regulation (Laborde et al., 2018).

More specifically, HRV refers to the variation between each consecutive heartbeat, described as the interval period between sequential R-R peaks in the QRS complex of an ECG measure (1996). The heart receives dual innervation from the sympathetic and parasympathetic nervous system, the former promoting a shortening of the interbeat interval whilst the latter prolongs it. Amongst the methods employed to quantify this construct, the most common measure is the frequency domain index whereby high frequency (HF) domain provides a reliable measure of vagal function (Thayer, 2009). Two additional methods for recording HRV are the Root Mean Square Successive Difference (RMSSD) which utilizes a time-domain, and the Respiratory Sinus Arrhythmia (RSA) measure which provides the value of HRV in synchrony with the respiratory cycle. High HRV is indicative of increased parasympathetic activity whereas low HRV indicates reduced parasympathetic activity and increased sympathetic activity.

In terms of mental health, low HRV has been linked with major psychological disorders such as depression and anxiety disorder (Kemp et al., 2010; Brunoni et al., 2013; Chalmers et al., 2014). Low HRV is also predictive of a first-time cardiovascular event, higher adverse outcomes after myocardial infarction, greater inflammation, and increased mortality (Thayer and Lane, 2007; Buccelletti et al., 2009; Hillebrand et al., 2013). In contrast to this, higher HRV has been found to be associated with better cognitive-behavioral function, including greater executive function, stress management, coping, and social engagement (Beauchaine and Thayer, 2015).

One psychophysiological attempt to provide a theoretical model of this association between HRV and mental as well as physical health, has been provided by Thayer and Lane (2000) through their work on the neurovisceral integration model (NIM). The model delineates an anatomical network of forebrain, brainstem, spinal cord, and the central autonomic network (CAN), which is suggested to be responsible for the integration of sensory-visceral, emotional, and cognitive information, and the subsequent implementation of regulatory actions (Benarroch, 1993). At the forebrain level, within the insular cortex, the anterior cingulate and amygdala, viscero-somatic afferent information is integrated with multi-source inputs of motivational value. Concurrently, nuclei of the hypothalamus integrate autonomic and endocrine inputs to regulate homeostatic functions. At the brainstem level, within the periaqueductal gray matter, pain and stress-related information are also integrated, whereas at the parabrachial nucleus, the nucleus solitarius and medullary reticular formation, the reflexive control of visceral systems takes place.

Sensory information is mostly processed in a sequential-hierarchical fashion through the structures of the CAN, before reaching complete integration and motor output (Riganello et al., 2018). Monitoring the regulatory processes within the CAN are cortical areas, mainly led by the prefrontal cortex. Prefrontal control is essential in order to adaptively regulate self-regulatory responses based on environmental demands (Smith et al., 2017). The NIM posits focus on these cortical processes for the determining of emotional regulation. The inability to exert cortical control has been found to underly inflexible and dysregulated emotional responses (Park and Thayer, 2014). The primary autonomic output resulting from CAN integration takes place at the sinoatrial node of the heart, therefore, HRV can be utilized as an index of these functions. HRV represents the interplay between sympathetic and parasympathetic activity where it has been suggested that greater parasympathetic activity is indicative of effective cortical-inhibitory-control and self-regulation (Jennings et al., 2015).

The second major theory explaining the vagal circuit system is the polyvagal theory (PVT) (Porges, 1991). The PVT describes the development of the ANS from an evolutionary and adaptive perspective. It is theorized that the ventral myelinated aspect of the vagus nerve (which developed evolutionarily most recently) developed in order to promote productive social affiliations (Porges, 2007b). In doing this, when safe and calm environments are detected, the vagus nerve inhibits primitive defense reflexes of the sympathetic system in favor of metabolically more efficient and soothing functions. When this soothing “social-engagement system” is active there is often an increase in efficient situational judgement, flexible behavioral responses and pro-social traits such as the distension of facial muscles, modulation of speech tone, and greater reception of voices (Lucas et al., 2018). These effects can facilitate individuals into organized societies and communitarian structures, providing an advantage for the survival of the species (Porges and Center, 2018).

The PVT introduces the novel concept of neuroception, which is the subconscious determination of environmental danger as a result of the integration of interoceptive, somatosensory, and endocrine information (Porges, 1991). Visceral interoceptive information is largely conveyed by the afferent fibers of the vagus nerve and subsequently integrated within higher relay areas to produce emotional and behavioral responses. Altered interoceptive pathways may, therefore, cause abnormal neuroceptive states, inflexible emotional responses, and aberrant vagal outputs (Porges, 2017). The ventral area of vagus functions as a constant “brake” over more primitive sympathetic reflexes so that at rest the “vagal brake” is in full function, and is “released” when external and internal stimuli require larger mobilization of energy via the sympathetic nervous system (SNS) (Porges, 2011).

High SNS activity is associated with emotions such as fear and anger, and behaviors directed toward safety and protection which inhibit the promotion soothing, regulatory, functions. When the SNS exhibits long-term dominance over the peripheral nervous system (PNS), individuals are vulnerable to high stress levels and have detrimental effects both at psychological and physical dimensions. This SNS dominance occurs in cases, for example, of chronic pain and post-traumatic stress disorder (PTSD) (Williamson et al., 2015; Kolacz and Porges, 2018). Porges (2011) also defines a third dimension of the vagal system represented by the dorsal vagal complex (DVC). The DVC is activated during situations of extreme danger and mediates shutdown of all autonomic responses (i.e., the freezing response), which explains, for instance, the loss of bladder and bowel control in frightening situations. Porges suggested that the DVC is of paramount importance in the study of trauma such as PTSD, as it explains the protracted defensive behaviors people display after traumatic events.

## Aims and Objectives

Given the psychophysiological evidence outlined in the introduction that both interoception and vagal tone (heart rate variability) seem to have important functional roles for emotional regulation, the primary aim of this study was to produce a systematic review to explore and synthesize (see Method section) the available literature which has identified interoception and heart rate variability as predictors (associations) for emotional regulation. These are central regulatory functions (Ceunen et al., 2016; Holzman and Bridgett, 2017) which tend to be studied independently of one another. Although interoception and HRV are two relatively new fields of research, there is much evidence linking these systems both physiologically, such as described through PVT, as well as psychologically such as their shared impact on many aspects of health and disease both central pathology (e.g., anxiety and depression) and peripheral (e.g., chronic pain) (Buccelletti et al., 2009; Di Lernia et al., 2016). So, this systematic review addresses a key gap in the current literature, that is the identifying whether HRV and interoception predict emotional regulation outcomes and strategies.

The specific primary question being asked is: are interoception and HRV predictors (causally associated) for ER in adult healthy and clinical populations? In this case, the predictors interoception and HRV are the independent variables (see Methods section for specific indices included), whilst emotional regulation is the dependent variable (again see Methods section for the indices included). The secondary questions are as follows: Which types of ER strategies would yield a positive metal well-being outcome? Whether HRV or interoceptive indices predict these well-being outcomes or types of ER strategies employed?

The findings from this systematic review could, at a later date, facilitate the development of a unified model of these central regulatory systems to help develop more appropriate clinical diagnostics and therapeutic interventions.

## Methods

This systematic review followed the Systematic Reviews and Meta-Analyses (PRISMA) statement (Moher et al., 2009) to ensure transparent and comprehensive reporting of the methods and results.

### Search Strategy

Electronic-only databases were searched for this review. The selection for this included PsychINFO, Web of Science, PubMed, CINAHL, and MEDLINE. The specific search strings are presented in Table 1. Searches in the Boolean format were as follows: interocept^*^ AND heart rate variability AND emotion^*^. These terms were used to capture the majority of the research relevant to these areas. Searchers were conducted on all fields which includes the title, abstract and the main body of the text. In addition to this, other relevant known papers by the authors were included.

**Table 1:** Search terms used for each database.

**Database**	**Search string**
PubMed	[(interocept^*^) AND heart rate variability] AND emotion^*^ regulation
PsychInfo	(interocept^*^ AND heart rate variability AND emotion^*^)
Web of Science	TS = (interocept^*^ AND heart rate variability AND emotion^*^)
CINAHL	(interocept^*^ AND heart rate variability AND emotion^*^)
MEDLINE	(interocept^*^ AND heart rate variability AND emotion^*^)

### Screening

All of the papers were assessed independently by two reviewers TP and DE by title and abstract initially. Once the relevant papers were selected and the duplicates were removed (see PRISMA Table in Results section), the remaining studies were examined in full text format by both TP and DE. Critical appraisal (see Critical Appraisal and Risk of Bias Assessment section) and final selection were assessed by TP and DE independently. In cases where there were discrepancies, the reviewers discussed these until consensus was made.

### Inclusion and Exclusion Criteria

As recommended by the Cochrane Handbook for systematic reviews, the PICO framework was utilized to define inclusion and exclusion criteria, and the research question (Higgins and Green, 2011). PICO is an acronym for population (sample type), intervention, comparator (what an intervention is compared to), outcome (what are the outcome measures). As this study did not explore specific interventions or comparators only the population and outcome were identified in the inclusion and exclusion criteria, along with type of study design.

### Population

Only human participants were included, animal studies were excluded. Mixed-age adult-only populations were included, whilst study samples comprising of children, adolescent, and elderly populations were excluded as their anatomical, physiological, and psychological characteristics differ greatly from that of the “normal” general population (Banerjee and Chaudhury, 2010). Non-clinical, and clinical study samples were included, however, it was acknowledged that non-clinical samples reduce the number of confounding variables and more accurately represent the general population (Breakwell and Smith, 2012), though clinical samples provide an overview for predictors relating to clinical dysfunction specifically. Clinical populations were only considered if subjects were affected by Axis 1 disorders, which have a clear relationships with emotion regulation (Bradley et al., 2011). Individuals with intellectual disabilities and terminal disorders were excluded as the links between those associated psychological states and aspects of emotion regulation are less defined (Mcclure et al., 2009).

### Type of Study

In order to be included, studies needed to be peer reviewed and published in English. Commentaries, books, and dissertations were excluded. Studies involving pharmacological therapies and transcranial brain stimulation, which are out of the scope of this review were also excluded. Clinical and non-clinical randomized controlled trials (RCT) and observational studies were included so as long as these studies had some focus on associations (predictors) between either interoception (independent measure; IV) or heart rate variability (IV) (or bother of these), and emotional regulation (dependent measure; DV). Predictor analysis refers to regression-based studies, however, correlational studies were also included (in a minority of cases), though not strictly predictors, as though they cannot infer causality, they are useful in terms of identifying simple relations between variables. Systematic reviews and meta-analysis studies were not included.

It is important to understand the key differences between the types of studies, as they relate to bias. RCTs examine the efficacy of an intervention on a defined outcome, whereas observational studies are useful for understanding physiological/pathophysiological processes, making predictions, and establishing the strength of association between variables (Faraoni and Schaefer, 2016). Observational studies differ from RCTs as they do not include a strict protocol (RCTs follow CONSORT) such as including a control group, a randomization process, and predefined endpoints, granting wider freedom of enquiry but also greater vulnerability to bias (Sørensen et al., 2006). Two primary types of observational studies are cohort and cross-sectional studies. Cohort and case-control designs allow observations to be made over time, whilst cross-sectional studies report results relating to a specific point in time (Song and Chung, 2010).

### Outcome (Association Measures)

The predictor measures (IVs) of interest relate to that of interoception and HRV, whilst the DV (what is being predicted) relates to emotion regulation. Interoception, in this systematic review was indexed as Interoceptive Awareness (IA), Interoceptive Accuracy (IAc), Interoceptive sensibility (ISen), as already defined in the literature (Garfinkel and Critchley, 2013; Forkmann et al., 2016).

Recent work by Garfinkel and Critchley (2013) has suggested that there is a dissociation between these indices in how they are measured and what they predict (Garfinkel et al., 2015). In order to reduce bias across measures, only interoceptive studies which utilized an objective measure of heart rate awareness were selected in this study (i.e., IA and IAc), whereas subjective measures were excluded (ISen). More specifically, only studies which used the mental tracking methods first developed by Schandry (1981) and later slightly modified versions (which often include more trials), were selected. Other objective methods such as Whitehead Heartbeat Discrimination Tasks (Schulz et al., 2013) were excluded from this study in order to reduce bias, as these have been suggested to relate to different underlying processes (Schulz et al., 2013; Garfinkel et al., 2015).

Heart rate variability was indexed by the commonly implemented means of high-frequency domain indices (HF), root mean square of the successive differences (RMSSD), and respiratory sinus arrhythmia (RSA). In some cases, some studies formulated bespoke indices for these (where these are included, they are defined). For both HRV and interoception, participants needed to have been recorded with a heart rate monitor (a pulse monitor or ECG for interoception and an ECG monitor for HRV).

Emotion regulation was index through commonly used and validated measures. This included the emotion regulation questionnaire (ERQ) (Gross and John, 2003) which comprises 6 items measuring reappraisal and 4 items measuring suppression. The ERQ has demonstrated good internal consistency for both subscales and is considered the gold standard measure for ER (Enebrink et al., 2013). Other commonly utilized measures for ER which were included were the difficulties in emotion regulation scale (DERS) (Gratz and Roemer, 2004); the spontaneous affect regulation scale (SARS) (Egloff et al., 2006); the cognitive emotion regulation questionnaire (CERQ) (Garnefski et al., 2001), which utilizes emotion-evoking visual tasks and daily diary reports; and the UWIST Mood Adjective Checklist, which provides a list of mood adjectives (e.g., happy, dissatisfied etc.) to assess the current mood of the individual (Matthews et al., 1990).

### Critical Appraisal and Risk of Bias Assessment

As the studies in this review were based on (prediction) regression and correlational analysis, The Checklist for Critical Appraisal and Data Extraction for Systematic Reviews of Prediction Modeling Studies (CHARMS) was utilized. This is a tool developed by the Cochrane Collaboration Prognosis Review Method Group (Moons et al., 2014) to critically apprise the quality of reporting and bias. This was used as opposed to the Transparent Reporting of a Multivariable Prediction Model for Individual Prognosis or Diagnosis (TRIPOD) and Prediction Model Risk of Bias Assessment Tool (PROBAST) assessments, which, instead, specialize in assessing developing, and updating prediction models (Debray et al., 2017). CHARMS has 10 domains of inquiry, however, two of these sections were not relevant. Specifically, “model evaluation” and “performance” were removed as the studies explored did not utilize model validation assessments. Therefore, the CHARMS framework was adapted and included only the eight relevant domain sections (see Table 2 for this adapted CHARMS version). Table 3 shows the risk of bias quality appraisal assessments made. All of the papers were assessed independently by two reviewers TP and DE, then where there were discrepancies the reviewers discussed these until consensus was met. Though there are no formal guidelines for a cut off criteria in the CHARMS literature (evaluation for this is made on a case by case basis), for the purposes of this systematic review, the criteria for inclusion was arbitrarily set through a simple dichotomous split, where papers would have to meet at least 50% of the criteria set by CHARMS in order to be included in the final synthesis of studies (all studies were included).

**Table 2:** Adapted relevant eight domains of the CHARMS checklist criteria.

**Source of the data**
Participants
Outcomes to be predicted
Candidate predictors
Sample size
Model development (type of model)
Results
Interpretation and limitations

**Table 3:** Critical appraisal using CHARMs tool for assessing risk of bias.

**References**	**Source of data (specified study design, e.g., cohort, cross-sectional, RCT etc.)**	**Participant details**	**Outcomes to be predicted**	**Candidate predictors**	**Sample size and missing data**	**Model development (type of model)**	**Results (correct presentation)**	**Interpretation and limitations**
Geisler et al. (2010)	Unclear	Low	Low	Low	Low	Low	Low	Low
Williams et al. (2015)	Unclear	Unclear	Low	Low	Low	Low	Low	Low
Koval et al. (2013)	Unclear	Low	Low	Low	Low	Low	Low	Low
Aldao et al. (2016)	Unclear	Low	Low	Low	Low	Low	Low	Low
Stange et al. (2017)	Unclear	Low	Low	Low	Low	Low	Low	Low
Kever et al. (2015)	Unclear	Low	High	High	Low	High	Low	Low
Füstös et al. (2012)	Unclear	Low	High	High	Low	High	Low	Low
Pollatos et al. (2015)	Unclear	Unclear	Low	Low	Low	Low	Low	Low

*Low, Low risk of bias; High, High risk of bias; Unclear, Risk of bias is unclear*.

## Results

**Figure 1:**
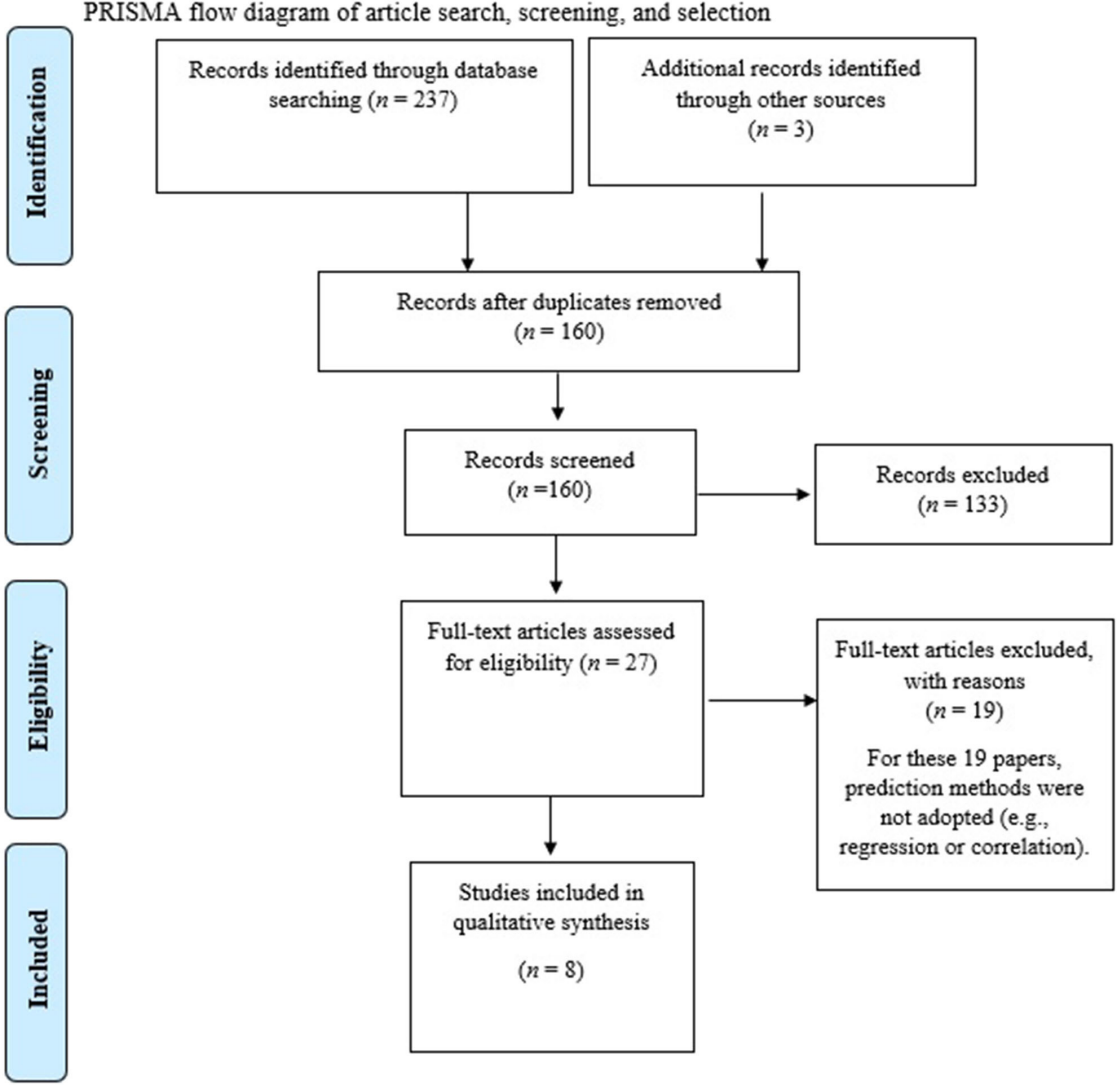
PRISMA flow diagram of article search, screening, and selection.

There were 237 papers initially identified through the databases, 79 through Web of Science, 24 through PubMed, 67 through PsychInfo, nine through CINAHL and 58 through MEDLINE. Three additional papers were identified outside of the initial search. After duplicates were removed 160 articles were screened through their title and abstract. After screening selection, 27 papers were then examined in full text. After critical appraisal analysis and full text assessment for eligibility, eight papers were selected for full analysis (see PRISMA Figure 1, and Table 4 for the summary of results).

**Table 4:** Summary of findings—relevant articles selected in final included results.

**References**	**Study focus on Interoception or HRV**	**HRV or Interoception index**	**Emotional regulation measures**	**Prediction model used**	**Results**
Geisler et al. (2010)	• HRV	• HF	• Cognitive emotion regulation questionnaire	• Linear regression	• Participants with higher HRV employed greater executive ER functions • HRV was related to positive hedonic tone and positive tense arousal
Williams et al. (2015)	• HRV	• InRMSSD • RRS • HF	• Emotion regulation scale	• Linear hierarchical regression	• Association between HRV (InRMSSD) and the difficulties in emotion regulation scale (DERS) total scores • InRMSSD was significantly associated with scores obtained from the DERS subscales clarity and impulse
Koval et al. (2013)	• HRV	• rMSSD • HF	• Affective instability • ER strategies	Linear regression – multilevel random intercept model	• HRV had a negative association with positive affective (PA) instability • Negative association between rMSSD and positive affect instability • Both rMSSD and HR were negative associated with negative affect
Aldao et al. (2016)	• HRV	• Composite score from HF, mean square difference, percentage of the absolute differences between consecutive IBIs, that are >50ms, and cardiac vagal index	• ER strategies (avoidance)	• Linear regression–GEE analysis	• Spontaneous avoidance was predicted negatively by the HRV index • Spontaneous avoidance in response to disgust eliciting pictures was positively associated by an interaction between the HRV index with the anxiety and depression symptoms score
Stange et al. (2017)	• HRV	• HF • RSA	• Positive or negative affect scale (PANAS) • ER strategies—spontaneous affect regulation scale (SARS) – • To identify ER strategies used (reappraisal, distraction, or suppression)	• Linear regression	• Significant interaction between reappraisal and recovery of HRV, whereby reappraisal predicted greater improvements in NA among those with higher HRV • Interaction between reappraisal and recovery, whereby reappraisal predicted greater improvements in NA for those with higher RSA recovery • Interaction between RSA reactivity and distraction, whereby distraction predicted improvements in NA but only for those with higher RSA reactivity to the sad film • interaction between distraction and recovery of HRV, whereby distraction predicted improvement in NA for those with greater HRV recovery • Suppression associated with increases in NA during recovery for those with lower HRV reactivity to the sad film
Kever et al. (2015)	• Interoception	• Interoceptive sensitivity	• Emotional regulation questionnaire • ER strategies—reappraisal, maintain	Correlation	• IS was higher in participants scoring high in the ERQ • This correlation was the same between IS and those employing greater reappraisal and supression strategies
Füstös et al. (2012)	• Interoception	• Interoceptive awareness	• Emotional arousal • ER strategies—reappraisal, maintain	• Correlation	• Found higher IA was positively correlated with emotional arousal during the negative maintain • When participants passively observing the negative valence images were asked to apply reappraisal ER techniques in the negative reappraisal (NR) condition, IA negatively correlated with emotional arousal • The downregulation in arousal between the passive-observing (NM) condition and the reappraisal (NR) condition (the difference between arousal in the two phases, NM-NR) was highly correlated with IA
Pollatos et al. (2015)	• Interoception	• Interoceptive sensitivity	• ER strategies—reappraisal, suppression • Emotional regulation questionnaire • Needs-Threat Scale	• Forward selection linear regression	IS predicted higher emotional regulation strategies; reappraisal and suppression

### HRV and Emotion Regulation

Three papers were identified which explored an association between HRV and emotional regulation. Of these, Geisler et al. (2010) found that participants with higher HRV (HF) had higher subjective well-being as indexed by the UWIST Mood Adjective Checklist. More specifically, HRV (IV) predicted (positively associated) positive hedonic tone (cheerfulness) (*p* < 0.05, adj. *R*^2^ = 0.02) and positive tense arousal (calmness) (*p* < 0.05, adj. *R*^2^ = 0.03), indicating that parasympathetic activity is associated with positive emotions. HRV also predicted the use of executive ER functions (as indexed by the Cognitive Emotion Regulation Questionnaire), and as a mediator (M) between HRV and subjective well-being (*p* < 0.01, adj. *R*^2^ = 0.04) but it did not predict the use of non-executive emotion regulation, (*p* = 0.54, adj. *R*^2^ = 0.00) (as a mediator between HRV and subjective well-being). In addition to this, HRV (IV) including executive emotion regulation as the mediator (M) predicted positive hedonic tone (DV), (*p* < 0.001, adj. *R*^2^ = 0.09), and positive tense arousal (DV), (*p* < 0.01, adj. *R*^2^ = 0.08). To extend the ecological validity of the results, the authors recorded HRV over a full day, confirming that HRV is associated with higher DERS scores over this longer period (*r* = −0.38, *p* = 0.049). In terms of life satisfaction, HRV (IV) was not related to past, present, and expected future satisfaction with life, (*p* > 0.25, adj. *R*^2^ < 0.01). Though HRV (IV) and executive emotion regulation (M) together predicted present satisfaction with life (DV) (*p* < 0.01, adj. *R*^2^ = 0.06), and expected future satisfaction with life (DV), (*p* < 0.001, adj. *R*^2^ = 0.09). This indicated that executive emotion regulation significantly mediated the influence of HRV on present and expected future life satisfaction. The authors concluded that HRV could be used as an index of emotional self-regulatory strength.

Williams et al. (2015) also found evidence which supported the idea that HRV and emotional regulation were associated. They used a hierarchical regression and found that HRV (InRMSSD; log transformed RMSSD) predicted (association) the outcomes of the difficulties in emotion regulation scale (DERS) total scores (r_partial_ = −0.222, *p* < 0.01) after controlling for age, ethnicity, gender, body mass index (BMI), Spielberger trait anxiety inventory (STAI-T) scores, ruminative response scale (RRS) scores, and HF peak (respiration) values. In addition to this, InRMSSD was significantly negatively associated with scores obtained from the subscales; emotional clarity (r_partial_ = −0.175, *p* < 0.05) and impulse control (r_partial_ = −0.155, *p* < 0.05). It is important to note that high scores of DERS indicate greater difficulties with emotion regulation, so that an inverse association indicates that high HRV is indicative of better emotional regulation. They concluded that their findings supported NIM.

Finally, Koval et al. (2013) explored the mechanisms which underpinned affective instability (mood fluctuations), and used trait HRV (indexed by RMSSD and HF) as a measure of emotion regulation capacity. Koval et al. used a regression multilevel random intercept model and reported that HRV predicted (negatively associated) positive affective (PA) instability (β = −0.18, *p* = 0.010 and β = −0.17, *p* = 0.006). Again, it is important to note an inverse association between HRV and affect instability indicates that high HRV is indicative of better emotional regulation. As the authors suggest, affective instability is likely to relate to faulty cortical inhibitory mechanisms central in ER. They also found an association between HRV (RMSSD) and positive affect instability both before (β = −0.16, *SE* = 0.08, *p* = 0.049) and after controlling for positive affect mean level (β = −0.16, *SE* = 0.08, *p* = 0.044). Similarly, there was some evidence that HR and positive affect instability were associated both before (β = −0.17, *SE* = 0.08, *p* = 0.045) and after controlling for PA mean level (β = −0.16, *SE* = 0.09. *p* = 0.061). In addition to this, after excluding outliers on each index of vagally mediated HRV and controlling for negative affect mean level, there was evidence that both RMSSD and HR were negatively associated with negative affect (NA) (RMSSD: β = −0.25, *SE* = 0.10, *p* = 0.017; HF: β = −0.32, *SE* = 0.12, *p* = 0.009). Finally, there was also some weak evidence to suggest that the two HRV indices were negatively associated with the standard deviation of PA (rMSSD: *r* = 0.23, *p* = 0.04; HF: *r* = 0.21, *p* = 0.06).

### HRV and Emotion Regulation Strategies

Three studies considered the association between HRV and ER strategies. Of these, Aldao et al. (2016) used a generalized estimation equation (GEE) regression analysis with a HRV index composed of a composite score of four measures (HF, mean square difference, percentage of the absolute differences between consecutive interbeat intervals that were >50 ms, and cardiac vagal index). They found that spontaneous avoidance in response to disgust eliciting pictures was predicted (negatively associated) by resting HRV index (Wald's χ^2^ = 4.415, b = −0.091, SE = 0.0435, *p* = 0.036). They also found that spontaneous avoidance in response to disgust eliciting pictures was positively associated by an interaction between the HRV index with the anxiety and depression symptoms score, where resting HRV is low and the symptoms are high (Wald's χ^2^ = 9.654, *b* = 0.192, SE = 0.062, *p* = 0.002). This meant that those participants with elevated symptoms of depression and anxiety whilst spontaneously using avoidance were higher in those with low HRV.

Based on these findings that high resting vagal tone (HRV) predicted lower use of spontaneous use of avoidance in response to disgust-eliciting pictures, and to a greater degree than anxiety and depression symptoms, they concluded that vagal tone may therefore be a protective factor against the use of avoidance based strategies in individuals experiencing elevated symptoms of anxiety and depression. They suggested this may have important implications with how to counteract affective disturbances which relate to avoidance such as rigid maladaptive strategies (e.g., avoidance) and underlies many mental health disorders today (Strosahl and Wilson, 1999; Aldao et al., 2010). They also suggested the findings highlighted the importance of using a HRV index as well as subjective measures when studying emotional regulation as the biological measure may be a more reliable predictor of emotion.

Complementing these findings, Stange et al. (2017) explored whether parasympathetic recovery (indexed by fluctuations in HF-HRV and respiration sinus arrhythmia; RSA) moderated associations between spontaneous regulation (ER strategies of reappraisal, distraction, or suppression) to predict affect recovery (improvements in negative affect; NA). They found a significant interaction between reappraisal and recovery of HRV, whereby reappraisal predicted greater improvements in NA among those with higher HRV (*t* = 3.86, *p* < 0.005) but not lower HRV (*t* = −0.11, *p* = 0.92). They also found an interaction between reappraisal and recovery, whereby reappraisal predicted greater improvements in NA for those with higher RSA recovery (*t* = 3.86, *p* < 0.05), but not for those will lower RSA recovery (*t* = 0.69, *p* = 0.49). For distraction, there was an interaction between RSA reactivity and distraction, whereby distraction predicted improvements in NA but only for those with higher RSA reactivity to the sad film (*t* = 2.39, *p* = 0.02) but not lower RSA reactivity (*t* = −0.83, *p* = 0.41). There was also an interaction between distraction and recovery of HRV, whereby distraction predicted improvement in NA for those with greater HRV recovery (*t* = 2.50, *p* = 0.01) but not for those with lower HRV recovery (*t* = −1.04, *p* = 0.30). For suppression, this was associated with increases in NA during recovery for those with lower HRV reactivity to the sad film (*t* = −2.36, *p* = 0.02) but not for higher HRV reactivity (*t* = 0.81, *p* = 0.42). They concluded by suggesting that HRV reactivity protected against the negative effects of suppression on NA recovery.

Koval et al. (2013) utilized two indices of vagally mediated HRV; a time domain measure in the form of rMSSD, and a frequency domain measure in the form of High Frequency component (HF, 0.15–0.40 Hz). They explored, through a multi-level regression, whether participant's daily affective instability was associated with certain “dysfunctional” ER strategies and HRV scores. The study was unable to show any significant association between affective instability and ER strategies such as reappraisal or suppression (measured with the ERQ), nor did HRV predict greater use of reappraisal or suppression.

### Interoception, Emotion Regulation, and Emotion Regulation Strategies

Three studies analyzed the relationships between interoception and emotion regulation. Kever et al. (2015) found that IS was higher in participants scoring high in the ERQ. This correlation was the same between IS and those employing greater reappraisal emotional strategies, as well as IS and those employing suppression strategies (*r* = 0.17; *p* = 0.001 for both). They concluded that higher detection of one's own bodily signals (higher interoception) facilitated the implementation of antecedent-focused as well as response focused emotional regulation strategies.

Füstös et al. (2012) analyzed the relationship between IA and emotional arousal in participants viewing pictures with negative valence and found higher IA was positively correlated with emotional arousal during the negative maintain (NM) condition (negative valence pictures but with no reappraisal attempted by the participants) (*r* = 0.38; *p* < 0.05). However, when participants passively observing the negative valence images were asked to apply reappraisal ER techniques in the negative reappraisal (NR) condition, IA negatively correlated with emotional arousal (*r* = −0.34, *p* < 0.05). Moreover, the downregulation in arousal between the passive-observing (NM) condition and the reappraisal (NR) condition (the difference between arousal in the two phases, NM-NR) was highly correlated with IA (*r* = 0.59, *p* < 0.01), which suggests that individuals with greater awareness of body-signals (greater interoception) were more effective at regulating emotions.

Finally, Pollatos et al. (2015) utilized a social-exclusion experiment (Cyberball paradigm) and measured emotional regulation through the ERQ through performing a regression analysis (forward selection). They found that IS predicted higher emotional regulation strategies; reappraisal (*t* = 3.14, β = 0.27, *p* < 0.01) and suppression (*t* = 3.22, β = 0.28, *p* < 0.01) scores for the exclusion condition. They concluded that IS can be associated with emotional regulation strategies and having awareness to bodily signals can help reduce aversive states.

## Discussion

This systematic review aimed to identify studies which have explored how HRV and interoception predict (associate with) ER and strategies. Interoception and HRV have been explored as biomarkers of health and well-being (such as positive emotional outcomes) previously, however, these have been largely explored independently, there has been no previous attempt to synthesize and compare these outcomes directly, and in the context of ER. They both seem to underlie the homeostatic system where interoception is thought of as the sensory component of the homeostatic system, whereas HRV indexes the primary output of the same homeostatic system in the form of the ANS (Craig, 2008; Park and Thayer, 2014).

The finding from this systematic review suggest that both interoception and vagal tone (HRV) have important functional roles for emotional regulation. The findings also show that some (non-avoidant) ER strategies were associated with better health and well-being, whilst (avoidant) ER strategies led to detrimental effects. More specifically, three of the included studies showed associations between high HRV and better emotion regulation (Geisler et al., 2010; Williams et al., 2015; Visted et al., 2017). One study highlighted the association between higher HRV and better executive-related ER skills, consequently generating better outcomes in mood and life-satisfaction (Geisler et al., 2010). These findings support NIM, as HRV, in that model, is suggested to be reflective of prefrontal-cortex activation which is the region associated with executive-function (Thayer et al., 2009; Fuster, 2015). Additional studies in the literature have demonstrated an association between high-HRV and better executive abilities such as inhibition, working memory, decision-making and general psychological flexibility, suggesting that this has applications for clinical research (Hansen et al., 2007, 2009; Hovland et al., 2012).

Two of the studies identified demonstrated negative associations between HRV and difficulties in regulating one's own emotions, as measured with the DERS (Williams et al., 2015; Visted et al., 2017). Visted et al. highlighted that non-accepting of one's own emotions (avoidance) prevents effective ER and was predicted by low HRV, whilst Williams et al. added strength to this association by controlling for covariates, anxiety, and rumination, and confirming the associations. Non-acceptance of emotions has been extensively linked with emotional inflexibility, leading to intensified emotional responses to stressors, general anxiety disorder, higher depressive symptoms and reduced overall well-being (Mennin et al., 2009; Ford et al., 2018). Conversely, acceptance is a foundational principle of several third-wave psychotherapeutic interventions which have emerged over the last few decades, such as Acceptance and Commitment Therapy (ACT) (Hayes et al., 2009). There is much evidence to suggest that what ACT postulates (acceptance of bad as well as good emotions and thoughts) is a necessary step in the path toward a flexible, purposeful, and values-based life (Hayes and Strosahl, 2004).

Three studies attempted to establish which ER strategy yielded the most positive outcomes (in relation to well-being) and whether HRV indices predicted these outcomes (Koval et al., 2013; Aldao et al., 2016; Stange et al., 2017). Aldao et al. (2016) and Stange et al. (2017) provided evidence of the negative impact of avoidance and suppression on psychological health, which were found to increase anxiety symptoms, depression, and negative affectivity in participants with lower HRV. It was not the same for participants with higher baseline HRV, who engaged in more adaptive ER strategies such as reappraisal. Within the more general literature, suppression has been predominantly linked with adverse psychophysiological outcomes, increased intensity of negative emotions and decreased positive emotionality and life satisfaction (Gross and Levenson, 1993; Gross and John, 2003). These findings were also supported by Stange et al. who demonstrated that people employing reappraisal and distraction strategies better coped with negative emotions and displayed higher HRV (Stange et al., 2017).

Not all of the evidence has supported a relation between ER strategies and emotional well-being. Koval et al. (2013), who collected data over seven consecutive days, found no significant association between suppression or reappraisal and daily affective instability (but they did find a negative association between HRV and positive affect instability). So, the evidence is not unanimously in support of the use of reappraisal over suppression strategies for ER. The benefits of reappraisal seem to be strictly context-dependent, as evidenced by Troy et al. (2013) who found reappraisal to be less adaptive at dealing with controllable stressors, as opposed to dealing with uncontrollable stressors. Moreover, cognitive reappraisal may lead to a heightened risk-taking and inhibit naturally occurring negative emotion (Heilman et al., 2010).

This alternative evidence is also supported by studies which showed that in Chinese populations, emotional suppression was shown to yield best psychological outcomes when compared with other ER strategies (Matsumoto et al., 2008; Soto et al., 2011; Nam et al., 2018). This highlights the need to explore cross-cultural differences as in nations with more rigid (less democratic) hierarchical and centralized societal-structures, emotion suppression may be more adaptive than reappraisal, whilst reappraisal may be more adaptive in libertarian western countries (Langner et al., 2012). Perhaps more flexible ways of regulating emotions, where one responds to situational demands, be it cultural, social, or physiological, in the pursuit of valued goals is important for mental well-being (Bonanno and Burton, 2013). HRV may be usefully applied as a biomarker of psychological and emotional flexibility (Gevirtz, 2015), and should be further explored in clinical settings.

Considerably less studies were identified which explored associations between interoception and ER, when compared to the literature on HRV. One reason may be that the research on interoception has traditionally focused on the neuroanatomical validation of the system, rather than exploring its wider psychosomatic implications (Ceunen et al., 2016). Secondly, the field is still relatively new, and still needs clear differentiation between the distinct facets of interoception, namely IAc, IA, and IS, as well as greater consensus on its heart beat measuring procedure (Kleckner et al., 2015; Forkmann et al., 2016).

Three studies recording baseline interoception were included in this review (Füstös et al., 2012; Kever et al., 2015; Pollatos et al., 2015). Kever and found that IS was greater in individuals applying either reappraisal or suppression strategies, with no statistical difference between the two, and lower in participants with overall reduced ERQ scores. Thus, IS seems to mediate greater ER regardless of the strategy employed by the participants. Furthermore, the results reported that females tend to utilize suppression more than males, highlighting gender differences in the selection of ER strategies. This highlights, again, the need to consider socio-anthropological factors, including socio-economic status and gender in the study of adaptive ER (Troy et al., 2017).

Füstös et al. (2012) showed that greater IA associated with higher emotional arousal when subjects viewed pictures with negative valence. Converging data from imaging studies has also demonstrated that greater interoceptive sensitivity is associated with greater sensitivity to emotional events (Pollatos et al., 2007). Moreover, it seems that increases in IAc drive short-term increases in sympathetic outputs, thus producing momentary reductions in HRV (Pollatos et al., 2012). In addition to this, in the same study by Füstös et al. (2012), when participants applied reappraisal ER to the negative pictures, those with higher IA were able to downregulate emotional arousal significantly better than subjects with lower IA.

Pollatos et al. (2015) demonstrated that people with higher IA were less prone to developing negative feelings when subjected to a social exclusion experiment (Cyberball paradigm), displaying flexible behaviors and ER strategies, as participants equally employed suppression and reappraisal. One expiation or these findings is that at the anterior insular, cingulate, and frontotemporal cortices, interoceptive information is integrated with auxiliary higher-order motivational inputs (Craig, 2009). This allows situational demands to be processed accordingly by executive functions (Garfinkel and Critchley, 2013; Adolfi et al., 2017), allowing for the flexible emotional strategies utilized.

In terms of implications, it is believed that interoceptive inputs are centrally integrated to produce meta-representations of “Self” necessary to mediate emotional responses. These, in turn, manifest via adaptive cognitive, behavioral, and autonomic responses. As the literature shows, dysfunctions within these systems may have pathological consequences both centrally (i.e., depression, anxiety, eating disorders) and peripherally (i.e., chronic pain) (Paulus and Stein, 2010; Klabunde et al., 2013; Di Lernia et al., 2016; Duschek et al., 2017). This work could help with indexing and diagnosing the psychophysiological etiology of the mental health dysfunction, combining with what is understood about HRV. For example, through understanding the individuals resting interoceptive and HRV states and their predicted (associated) outcomes, this may further inform practitioners about how to best to apply therapeutic techniques such as Cognitive Behavioral Therapy (CBT), mindfulness-based interventions, or ACT, which may interact with ER in different ways, thus improving patient-centered care.

Interoception has been linked with aspects of health and psychopathology whilst, concurrently, considerable evidence has explored the salient role of HRV, not only as a bio-marker of psychophysical health, but also as a direct modulator of central regulatory functions (Farb et al., 2015; Kemp et al., 2017). So, by targeting interventions which increase HRV via mind-body practices such as yoga, slow breathing and mindful meditation, beneficial changes can take place at central regulatory centers (Mather and Thayer, 2018), improving ER and ultimately well-being. Evidence has been mounting regarding the effectiveness of mind-body practices such as mindfulness at promoting restorative states alongside greater vagal activation (Sullivan et al., 2018; Zou et al., 2018), so these techniques are promising.

This work may also be considered complimentary to existing models of HRV and well-being. One example of this is the GENIAL (Genomics, Environment, vagus Nerve, social Interaction, Allostatic regulation, Longevity) model, which is behavioral, psychological and physiological model which identifies the vagus nerve indexed by HRV as an important mediator and moderator which influence social ties, subsequent health and well-being, as well as longevity (Kemp et al., 2017). Kemp et al. suggest that future research should initiate a multi-disciplinary focus to develop new lines of inquiry related to this. The findings from this systematic review, may therefore, help facilitate further expansion of the GENIAL model, with the incorporation of the predictive factors of interoceptive and emotional regulatory functions in the context of health and well-being. Such further development of the GENIAL model could help facilitate new diagnostic and prognosis tools in a clinical setting, as well as bespoke patient-centered interventions.

Of course, there are limitations with this study. First of all, the studies identified utilized homogeneous populations of, mostly, university students. Without longitudinal and population-based studies, it cannot be understood the exactly whether these findings can be generalized (Zwahlen and Salanti, 2018). Future studies should focus on longitudinal and population-based studies which incorporate both HRV and interoception in their design methodology. In addition to this, given the relatively small number of papers identified with association analyses between interoception and ER, correlational studies were included, but causality in these cannot be determined. Indeed, the fact that there are a limited number of papers identified in the final output selection means that any interpretations of these findings should be considered cautiously. More studies in the future with larger sample sizes and a greater variety of interoception as well as HRV indices should be considered.

Another potential limitation of this review was it did not identify the distinction between state and trait differences. These have been shown to exist for both HRV (Bertsch et al., 2012) and interoception (Wittkamp et al., 2018), and are typically indices which are stable (trait) or variable (state) over time. HRV and interoception state and trait studies explore temporal differences through latent state trait analysis (such as through repeated subject measures) as opposed to the largely cross-sectional studies explored in this review (only Koval et al. explored HRV as a trait through repeated measures).

These distinctions may be important to explore in the future as studies have suggested that when stable over time HRV, for instance, can be considered a trait characteristic, which can reflect a person's ability to adapt to situations (Thayer et al., 2009). So, the distinction between state and trait may be important when interpreting ER strategies to difficult situations (e.g., when adopting a psychologically flexible vs. avoidant strategy type). Unfortunately, there is simply not enough associational (prediction) studies available at present in order to draw distinctions between state and trait for this type of (prediction based) review. More studies in the future should explore state and trait differences specifically, and within the context of combined interoceptive and HRV associational studies.

A last potential limitation was that ER was assessed in a number of different modalities, comprising various questionnaires, attitudinal and cognitive-behavioral tasks. The lack of a standardized measurement procedure prevents precise comparisons between studies to made. Ideally, measuring methods should all assess the same aspect of ER and present an identifiable aspect of the task, a process defined as metrological traceability (Vesper et al., 2016).

In conclusion, HRV showed significant associations with better ER, largely predicting the use of reappraisal strategies over suppression. These findings are in line with the predominant trend of thought that regards reappraisal as the most adaptive strategy for regulating emotion. Studies considering the long-term regulation of emotions, cross-cultural, and gender differences in ER, however, seem to challenge this trend supporting the need for a flexible approach to emotions, that may involve either suppression or reappraisal dependent on the context. It is concluded here that high HRV should be considered as a biomarker of flexible ER. Despite the fewer studies scrutinizing interoception in ER, people with higher interoception scored higher on the ERQ, better handled negative emotions and feelings of social exclusion. These findings may provide evidence of bottom-up mechanisms of self-regulation and could help facilitate further adaptions on the existing GENIAL model. More research is needed to understand the specific causal order of influence between these constructs, and the peripheral interactions between interoceptive and parasympathetic systems. This may aid in the development of psychological interventions targeted toward these specific functions in a clinical setting and promote more bespoke and patient-centered care options.

## Author Contributions

TP designed this study with assistance from DE. TP and DE conducted the analysis equally and wrote the paper.

## Conflict of Interest

The authors declare that the research was conducted in the absence of any commercial or financial relationships that could be construed as a potential conflict of interest.
